# Molecular epidemiology of Porcine torovirus (PToV) in Sichuan Province, China: 2011–2013

**DOI:** 10.1186/1743-422X-11-106

**Published:** 2014-06-05

**Authors:** Lu Zhou, Haoche Wei, Yuancheng Zhou, Zhiwen Xu, Ling Zhu, Jim Horne

**Affiliations:** 1 Animal Biotechnology Center, College of Veterinary Medicine, Ya’an, China; 2 Key Laboratory of Animal Disease and Human Health, College of Veterinary Medicine, Sichuan Agricultural University, Ya’an, China; 3 Faculty of Bio and Biochemistry, University of Bath, Bath spa, England

**Keywords:** Porcine torovirus, Membrane gene, Phylogenetic analysis

## Abstract

**Background:**

Porcine torovirus (PToV) is a member of the genus Torovirus which is responsible for gastrointestinal disease in both human beings and animals with particular prevalence in youth. Torovirus infections are generally asymptomatic, however, their presence may worsen disease consequences in concurrent infections with other enteric pathogens.

**Methods:**

A total of 872 diarrheic fecal samples from pigs of different ages were collected from 12 districts of Sichuan Province in the southwest of China. RT-PCR was done with PToV S gene specific primers to detect the presence of PToV positive samples. M gene specific primers were used with the PToV positive samples and the genes were sequenced. A phylogenetic tree was constructed based on the M gene nucleotide sequences from the 19 selected novel Sichuan strains and 21 PToV and BToV M gene sequences from GenBank.

**Results:**

A total of 331 (37.96%, 331/872) samples were found to be positive for PToV and the highest prevalence was observed in piglets aged from 1 to 3 weeks old. Through phylogenetic inference the 40 PToV M gene containing sequences were placed into two genotypes (I & II). The 19 novel Sichuan strains of genotype I showed strong correlations to two Korean gene sequences (GU-07-56-11 and GU-07-56-22). Amino-acid sequence analysis of the 40 PToV M gene strains revealed that the M gene protein was highly conserved.

**Conclusions:**

This study uncovered the presence of PToV in Sichuan Province, and demonstrated the need for continuous surveillance PToV of epidemiology.

## Introduction

Porcine torovirus (PToV) is a member of the genus *Torovirus*, family *Coronaviridae*, and order *Nidovirales*[1]. Toroviruses have been detected in fecal samples from cows, horses, humans, pigs and turkeys suffering from diarrheal illness [2-7]. The first identified torovirus was isolated from a horse with diarrhea (equine torovirus, EToV, also called Berne virus) in 1972 [8].

To date, research on toroviruses has been limited because of difficulties associated with their propagation *in vitro*. In 1983, EToV was found to be able to adapt and grow in equine cells. As a consequence, much of what is known of torovirus at the molecular level has been based on EToV [9].

PToV particles were initially observed by electron microscopy from pig fecal samples. They were described as spherical, oval, elongated, or kidney-shaped enveloped viruses possessing a positive-sense, single-stranded, polyadenylated RNA genome of approximately 25–30 kb in length [10-12]. In 2013, a study reported that the genome was found to be 28,301 bp long [13], sharing 79% identity with bovine torovirus (BToV). It mainly consists of a replicase (20,906 bp) and structural genes: spike, S (4,722 bp); membrane, M (702 bp); hemagglutinin-esterase, HE (1,284 bp); and nucleocapsid, N (492 bp) [13-15]. Sequence data for PToV strains are based on the S, M, HE, and N genes; M is more conserved compared with other genes, sharing 98% amino acid sequence identity between the described PToVs M genes [13], which have only been reported in Spain and South Korea.

PToV infections have been detected in many countries, including the Netherlands, Canada, South Africa, the United States, Hungary, the UK and South Korea. Serum samples, obtained from farms in Spain and screened via the ELISA method indicated very high seroprevalence against PToV in some studies [16,17], while other studies showed low seroprevalence [18]. In this article, we investigate the infection and characteristics of PToV on the basis of nucleotide and amino acid sequencing and phylogenetic analysis of M genes, for PToV isolated from porcine fecal samples in Sichuan Province, China.

## Methods and materials

A total of 872 fecal samples were collected from pigs with diarrhea across different districts of Sichuan Province during the winter from 2011 to 2013. Most of the sampled piglets were 1 to 3 weeks old, with the reminder being 3 to 7 weeks old, 7 to 11 weeks old or over 11 weeks old. Antibiotic treatment was invalid in all sampled pigs. Samples were analyzed based on collection time and geographical location in either the east or west sections of Sichuan Province. A portion of each sample was homogenized for RNA extraction, and the remaining samples were stored at −70°C.

According to the manufacturer’s instructions, total RNA was extracted from fecal homogenate using *TRIzol* reagent (Life Technologies, Gaithersburg, MD, USA). Viral cDNA was synthesized from RNA using reverse transcriptase (M-MLV, Takara, Kyoto, Japan) according to the manufacturer’s instructions, and stored at −20°C until used in RT-PCR reactions.

The cDNA was screened by PCR using the following method we had established. A 451-bp fragment of the conserved region of the S gene was amplified with the forward primer (5' - ACCCCTGCCTGAGGTTTCYTT - 3'), and reverse primer (5' – AGCACGACGTTGTCTRCGTGT - 3’). Amplification was carried out in PCR buffer containing 200 mM of each dNTP, 10 pmol of each primer, 1.0 U Taq DNA polymerase (Promega, Madison, WI, USA), and 1.5 mM MgCl_2_, in a total volume of 40 μl. PCR was performed at 94°C for 2 min, followed by 30 cycles of amplification (94°C for 30 s, 57°C for 30 s, and 72°C for 30 s), and a final extension of 72°C for 7 min. The PCR product was resolved using 1% agarose gel electrophoresis, stained with ethidium bromide (Invitrogen, Carlsbad, CA, USA), and visualized under ultraviolet light with the Bio-Rad gel imaging system (Hercules, CA, USA). All specimens were also tested for the presence of porcine epidemic diarrhea virus (PEDV), transmissible gastroenteritis coronavirus (TGEV) and group A rotavirus (RVA) in terms of the methods described in previous studies [19,20]. The PCR and Nested PCR specific primers used are listed in Table 1.

**Table 1:** Oligonucleotide primers used for the detection of PEDV, TGEV and RVA in fecal samples obtained from pigs with diarrhea

**Target viruses**^**a**^	**Target genes**^**b**^	**Primer sequence, 5’-3’**^**c**^	**Size (bp)**	**Source or reference**
PEDV	S	F:TTCTGAGTCACGAACAGCCA	651	[19]
		R:CATATGCAGCCTGCTCTGAA		
TGEV	S	F:GTGGTTTTGGTYRTAAATGC	859	[19]
		R:CACTAACCAACGTGGARCTA		
RVA	VP6	F: AAAGATGCTAGGGACAAAATTG	308	[20]
		R: TTCAGATTGTGGAGCTATTCCA		
		nF:GACAAAATTGTCGAAGGCACATTATA	121	
		nR: TCGGTAGATTACCAATTCCTCCAG		

^a^PEDV: porcine epidemic diarrhea coronavirus; TGEV: Transmissible gastroenteritis coronavirus; RVA: group A rotavirus.

^b^ORF: S: spike protein; VP6: viral protein 6.

^c^F: Forward primer for RT-PCR; R: Reverse primer for RT-PCR.

nF: Forward primer for nested PCR; nR: Reverse primer for nested PCR.

PToV genomic cDNA was obtained from 19 positive samples as described above. The 702 bp fragment of the complete M gene was amplified with primers M1 (5' - ATGTTTGATACAAATTTTTGGCCTT - 3') and M2 (5' – CTACTCAAACTTAACA CTTGACAACTGC - 3'). PCR amplification was carried out as described above, and the PCR products were visualized using 1% agarose gel electrophoresis under ultraviolet light.

The PCR products were gel-purified using a Gel Extraction Kit (Tiangen Biotech, Beijing, China). The purified target fragments were ligated into a linear vector pMD19-T (Takara, Dalian, China), and the recombinant plasmids were transformed into *Escherichia coli* DH5α competent cells (Invitrogen). The identity of the constructs was confirmed by sequencing (Invitrogen).

Genetic distance was initially measured by pairwise comparisons of nucleotide sequences to reference PToV sequences available in GenBank using the Basic Local Alignment Search Tool (BLAST) (http://blast.ncbi.nlm.nih.gov/). Multiple alignments were achieved using the Clustal_W method of the MegAlign 5.01 program (DNASTAR Inc., Madison, WI, USA) [21]. The phylogenetic tree was constructed using the Neighbor-Joining method on Molecular Evolutionary Genetics Analysis (MEGA) software version 5.0 [22] and carried out using the Kimura 2-parameter model [23]. The transition/transversion bias (R) and substitution rates were estimated using MEGA 5.0. The substitution pattern and rates were estimated using the Kimura 2-parameter model, with nucleotide frequencies of A = 25.00%, T/U = 25.00%, C = 25.00%, and G = 25.00% [23]. For estimating ML- maximum likelihood values, a user-specified topology was used. The maximum log likelihood for this computation was −1680.662 and −2731.225. Codon positions included were 1st + 2nd + 3rd + Noncoding. All positions containing gaps and missing data were eliminated. There were a total of 702 positions in the final dataset. Evolutionary analyses were conducted in MEGA5 [24]. All analyses were based on PToV M gene sequences.

The novel PToV M nucleotide sequences have been submitted to NCBI GenBank and assigned accession numbers KF727566-KF727584. Other toroviruses full-length sequences for M gene were obtained from GenBank (http://www.ncbi.nlm.nih.gov/nuccore).

## Results

**Figure 1:**
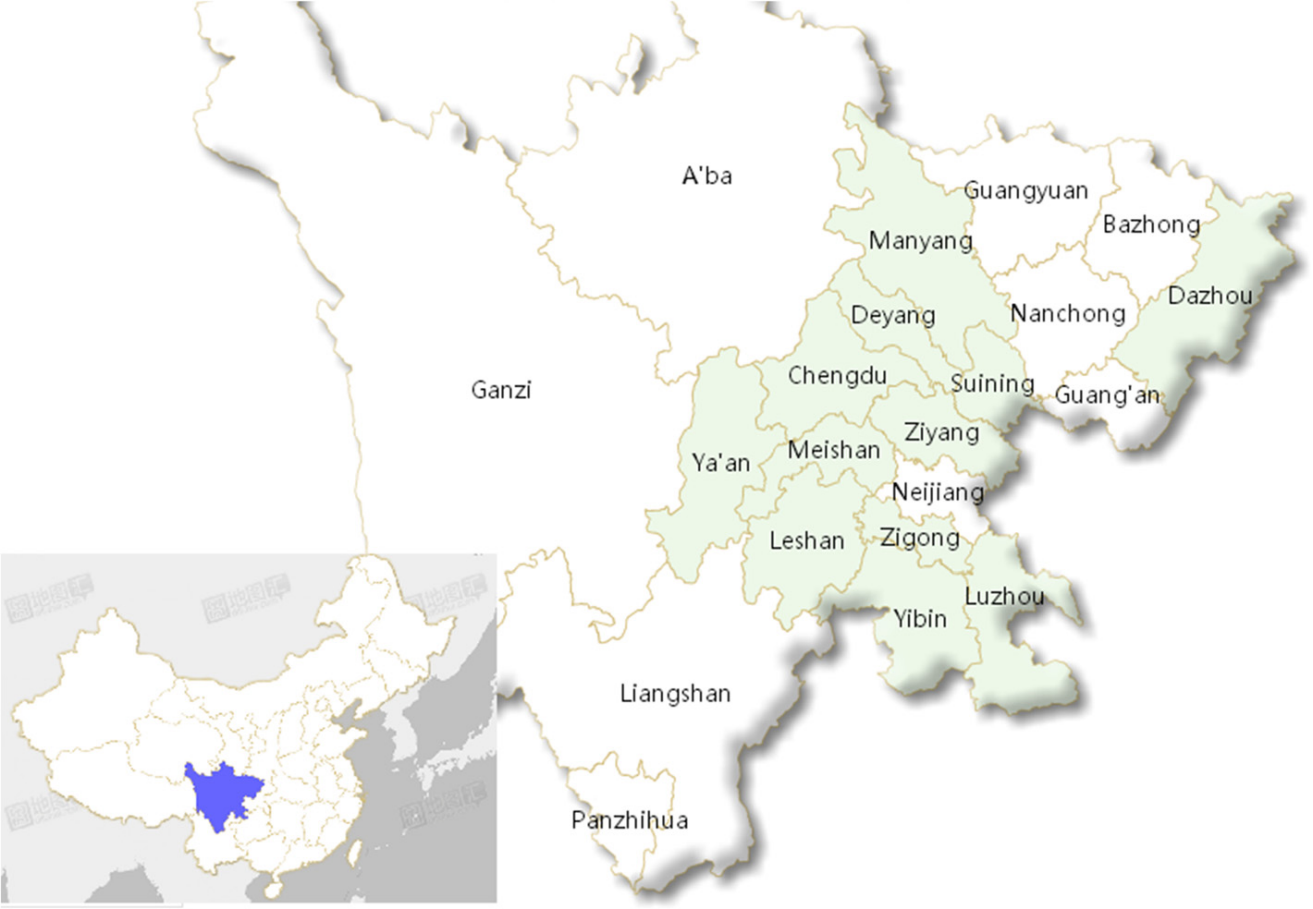
**Geographical locations of samples collected in this study.** The green parts indicate the districts of Sichuan Province where the porcine fecal samples were collected.

The samples from 12 districts in Sichuan Province, China are shown in Figure 1. Of these samples, 331 (37.96%, 331/872) were positive for PToV, as detected by RT-PCR. PToV infection rates were greater than 37.96% for Suining, Deyang, Yibin, Chengdu, Mianyang, and Meishan districts. PToV infection rates of 36.52% (42/115), 5.77% (3/52), 9.52% (2/21), 27.78% (10/36), 4% (1/25), and 10.26% (4/39) were detected for Ya’an, Leshan, Dazhou, Zigong, Luzhou and Ziyang, respectively. For other tested enteric pathogens, the positive rates of PEDV, TGEV, RVA were 62.5% (545/872), 12.5% (109/872) and 66.9% (583/872), respectively, while the PToV Co-infected with RVA, TGEV and PEDV positive rate was 4.1% (36/872). And the details of PToV, RVA, TGEV and PEDV infection and co-infection rates in different districts of Sichuan Province were in Table 2. The PToV positive rates at different growth stages of pigs revealed that 1 to 3 week old piglets have the highest PToV infection rate 42.47% (296/697), with PToV infection rates of 25.53% (24/94), 16.07% (9/56) and 8% (2/25) in 3 to 7 weeks, 7 to 11 weeks and over 11 weeks, respectively. The complete M sequence of 42 of the 331 PToV-positive strains were amplified and sequenced. Of the 42 PToV strains, 19 strains were selected, based on geographic factors and their isolation from different farms in the same district.

**Table 2:** Summary of enteric pathogens present in the fecal samples obtained from pigs with diarrhea

**District**	**Infection rate (%)**				**PToV and PEDV Co-infection**	**PToV and RVA Co-infection**	**PToV and TGEV Co-infection**	**PToV, RVA, TGEV and PEDV Co-infection**
	**PToV**	**PEDV**	**RVA**	**TGEV**				
Meishan	47.1%(64/136)	64%(87/136)	67.6%(92/136)	13.2%(18/136)	47.1%(64/136)	47.1%(64/136)	12.5%(17/136)	3.7%(5/136)
Leshan	5.77%(3/52)	71.1%(37/52)	78.8%(41/52)	15.4%(8/52)	5.77%(3/52)	5.77%(3/52)	15.4%(8/52)	3.8%(2/52)
Mianyang	51.9%(69/133)	74.4%(99/133)	73.7%(98/133)	15%(20/133)	50.4%(67/133)	48.9%(65/133)	15%(20/133)	6%(8/133)
Ya’an	36.5%(42/115)	76.5%(88/115)	79.1%(91/115)	0	36.5%(42/115)	31.3%(36/115)	0	0
Dazhou	9.5%(2/21)	52.4%(11/21)	61.9%(13/21)	0	0	9.5%(2/21)	0	0
Ziyang	10.3%(4/39)	66.7%(26/39)	64.1%(25/39)	10.3%(4/39)	10.3%(4/39)	10.3%(4/39)	7.7%(3/39)	5.1%(2/39)
Yibin	38.9%(21/54)	0	63%(34/54)	13%(7/54)	0	35.2%(19/54)	13%(7/54)	0
Chengdu	48.8%(40/82)	67.1%(55/82)	81.7%(67/82)	18.3%(15/82)	47.6%(39/82)	43.9%(36/82)	14.6%(12/82)	4.9%(4/82)
Deyang	41.8%(43/103)	74.8%(77/103)	69.9%(72/103)	15.5%(16/103)	26.2%(27/103)	32%(33/103)	15.5%(16/103)	7.8%(8/103)
Luzhou	4%(1/25)	0	0	0	0	0	0	0
Suining	42.1%(32/76)	63.2%(48/76)	65.8%(50/76)	25%(19/76)	35.5%(27/76)	42.1%(32/76)	25%(19/76)	9.2%(7/76)
Zigong	27.8%(10/36)	47.2%(17/36)	0	5.6%(2/36)	22.2%(8/36)	0	0	0

**Figure 2:**
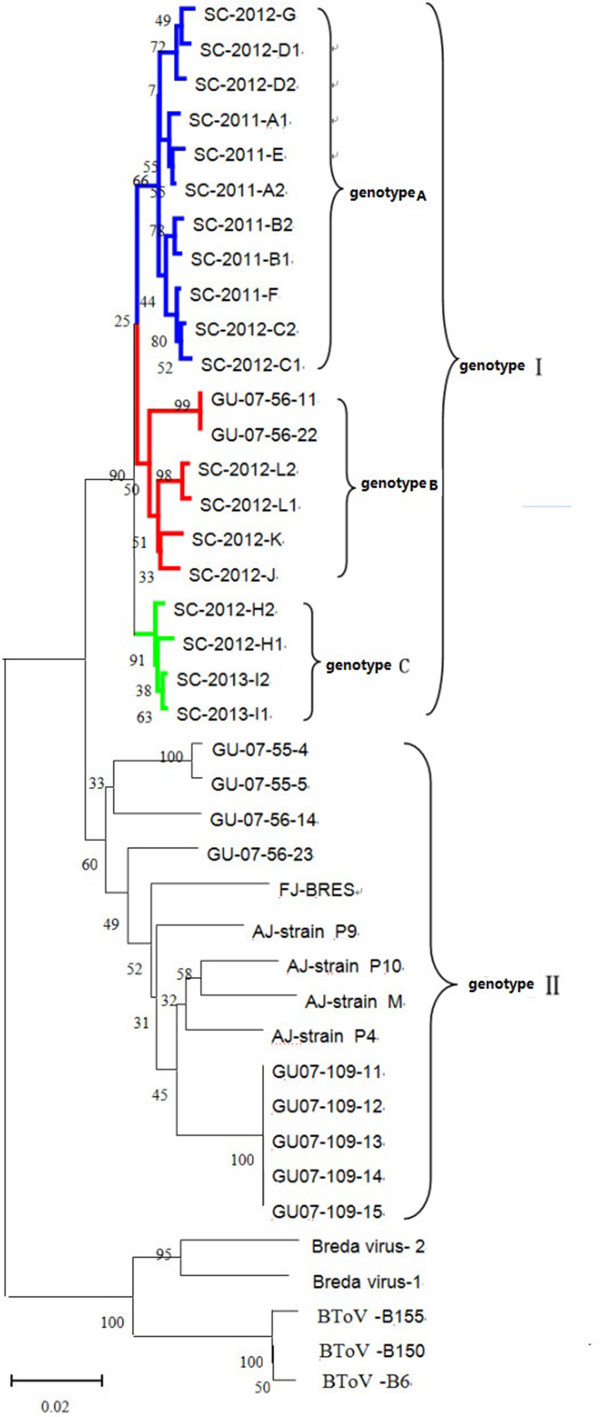
**Phylogenetic analysis based on the complete nucleotide sequence (702 bp) of gene M for novel PToV isolated from Sichuan, China, and reference viruses (GenBank).** The reference PToV M nucleotide sequences were obtained from GenBank: Korea strain (accession no. GU181240.1, GU181241.1, GU181242.1, GU181243.1,GU181244.1, GU181245.1 ,GU181246.1, GU181247.1, GU181248.1, GU181249.1, GU181250.1); Netherlands strain(accession no. AJ575374.1, AJ575376.1, AJ575377.1, AJ575369.1, AJ575368.1, AJ575371.1); Canada strain (accession no. AF076621.1, AY427798.1); The 19 strains analyzed in this study are named by SC-.

The complete M gene sequences of the 19 strains were compared with each other, and against reference sequences for 21 strains of toroviruses in the GenBank database (Table 3). As shown in Figure 2, phylogenetic and homology studies at the nucleotide (702 bp) level demonstrated that PToV strains belonged to two genotypes (I and II), separated with bootstrap values of 90 and 60, respectively. All 19 novel PToV strains within genotype I were further divided into three genotypes (A, B and C). With only two exceptions (GU-07-56-11 and GU-07-56-22), all other representative strains from NCBI belonged to genotype II. Strains GU-07-56-11 and GU-07-56-22 shared high homology with four novel PToV strains, SC-2012-L1, SC-2012-L2, SC-2012-K and SC-2012-J, and were classified as genotype B. Nucleotide and the deduced amino acid sequence homologies for the 19 Chinese PToV M sequences identified in this study were between 97.3–99.9% and 99.1–99.6% respectively. The Chinese PToV strains were 92.7–99.9% and 97.4–99.6% identical to the PToV strains in other countries while only 80.1%–82.8% and 89.3%–91.0% to those of bovine torovirus.

**Table 3:** GenBank accession numbers of Chinese PToVs and the reference toroviruses used in molecular and phylogenetic analyses”

**No.**	**Name**	**Isolation year**	**Area**	**Accession no.**	**No.**	**Name**	**Isolation year**	**Area**	**Accession no.**
1	BToV -B150	2003	Netherlands	AJ575376	21	FJ-BRES	2009	Spain	FJ232069
2	BToV -B155	2003	Netherlands	AJ575377	22	SC-2011-B1	2011	Mianyang	KF727568
3	AJ-strain_P4	2003	Netherlands	AJ575369	23	SC-2011-B2	2011	Mianyang	KF727569
4	AJ-strain_P9	2003	Netherlands	AJ575370	24	SC-2011-E	2011	Dazhou	KF727574
5	AJ-strain_P10	2003	Netherlands	AJ575371	25	SC-2011-A1	2011	Meishan	KF727566
6	BToV -B6	2003	Netherlands	AJ575374	26	SC-2011-A2	2011	Meishan	KF727567
7	AJ-strain_M	2003	Netherlands	AJ575368	27	SC-2011-F	2011	Ziyang	KF727575
8	Breda virus-1	2005	Canada	AF076621	28	SC-2012-L1	2012	Ya’an	KF727579
9	Breda virus-2	2005	Canada	AY427798	29	SC-2012-L2	2012	Ya’an	KF727580
10	GU07-109-15	2009	Korea	GU181240	30	SC-2012-C1	2012	Chengdu	KF727570
11	GU07-109-14	2009	Korea	GU181241	31	SC-2012-C2	2012	Chengdu	KF727571
12	GU07-109-13	2009	Korea	GU181242	32	SC-2012-D1	2012	Deyang	KF727572
13	GU07-109-12	2009	Korea	GU181243	33	SC-2012-D2	2012	Deyang	KF727573
14	GU07-109-11	2009	Korea	GU181244	34	SC-2012-H1	2012	Yibin	KF727577
15	GU-07-56-23	2009	Korea	GU181245	35	SC-2012-H2	2012	Yibin	KF727578
16	GU-07-56-11	2009	Korea	GU181248	36	SC-2012-K	2012	Luzhou	KF727582
17	GU-07-55-5	2009	Korea	GU181249	37	SC-2012-G	2012	Suining	KF727576
18	GU-07-55-4	2009	Korea	GU181250	38	SC-2012-J	2012	Zigong	KF727581
19	GU-07-56-14	2009	Korea	GU181247	39	SC-2013-I1	2013	Leshan	KF727573
20	GU-07-56-22	2009	Korea	GU181246	40	SC-2012-I2	2013	Leshan	KF727574

The 19 novel M nucleotide sequences and their deduced amino acid sequences were aligned. The estimated transition/transversion bias (R) was 1.15 among the 19 PToV strains from Sichuan in China, and 1.84 among global PToV strains. The Sichuan PToV nucleotide sequences presented 54 mutations (Table 4); 26 of which were commonly mutated sites (at nucleotide positions 17, 18, 27, 39, 72, 121, 276, 288, 306, 495, 512, 513, 537, 552, 561, 579, 618, 633, 636, 652, 654, 657, 663, 666, 678, 690) in the gene M nucleotide sequence. The Sichuan strains had three common amino-acid substitutions(Y^6^ → F, N^171^ → S, N^181^ → S), while the rest were silent mutations.

**Table 4:** Comparison of nucleotide and deduced amino acid sequences of M genes between 19 Chinese PToVs and reference PToV (GenBank accession No. GU181244)

**District**	**Name**	**Nucleotide substitutions**																									
		**1**	**1**	**2**	**3**	**7**	**9**	**1**	**1**	**1**	**1**	**1**	**2**	**2**	**2**	**2**	**3**	**3**	**4**	**4**	**4**	**4**	**4**	**4**	**4**	**5**	**5**
		**7**	**8**	**7**	**9**	**2**	**3**	**1**	**2**	**2**	**5**	**7**	**2**	**2**	**7**	**8**	**0**	**8**	**0**	**2**	**3**	**4**	**4**	**4**	**9**	**1**	**1**
								**1**	**1**	**3**	**1**	**2**	**2**	**8**	**6**	**8**	**6**	**4**	**8**	**3**	**2**	**1**	**2**	**7**	**5**	**2**	**3**
Meishan	SC-2011-A1	T	T	C	A	A	C	-	C	T	C	-	T	-	T	G	C	C	-	C	-	-	C	-	T	G	C
	SC-2011-A2	T	T	C	A	A	C	C	C	T	C	T	T	-	T	G	C	C	-	C	-	-	C	-	T	G	C
Mianyang	SC-2011-B1	T	T	C	A	A	C	C	C	C	C	T	T	-	T	G	C	C	-	C	-	-	C	-	T	G	C
	SC-2011-B2	T	T	C	A	A	C	C	C	T	C	T	T	-	T	G	C	C	-	C	-	-	C	-	T	G	C
Dazhou	SC-2011-E	T	T	C	A	A	C	C	C	T	C	T	T	-	T	G	C	C	-	C	-	-	C	-	T	G	C
Ziyang	SC-2011-F	T	T	C	A	A	C	C	C	T	C	T	T	C	T	G	C	C	-	C	-	-	C	-	T	G	C
Chengdu	SC-2012-C1	T	T	C	A	A	-	C	C	T	-	T	T	-	T	G	C	C	-	C	-	-	C	-	T	G	C
	SC-2012-C2	T	T	C	A	A	C	C	C	T	-	T	T	C	T	G	C	C	-	C	-	-	C	-	T	G	C
Deyang	SC-2012-D1	T	T	C	A	A	C	C	C	C	C	-	T	C	T	G	C	C	T	-	-	-	C	-	T	G	C
	SC-2012-D2	T	T	C	A	A	C	C	C	C	C	-	T	C	T	G	C	C	T	-	-	-	C	-	T	G	C
Suining	SC-2012-G	T	T	C	A	A	C	C	C	C	C	-	T	C	T	G	C	C	T	-	-	-	C	-	T	G	C
Zigong	SC-2012-J	T	T	C	A	A	C	C	C	C	C	T	-	-	T	G	C	-	T	C	-	-	C	-	T	G	C
Luzhou	SC-2012-K	T	T	C	A	A	C	C	C	T	C	T	-	-	T	G	C	-	T	C	T	A	C	T	T	G	C
Yibin	SC-2012-H1	T	T	C	A	A	C	-	C	T	C	-	-	-	T	G	C	-	-	-	T	-	C	-	T	G	C
	SC-2012-H2	T	T	C	A	A	C	-	C	T	C	-	-	-	T	G	C	-	-	C	T	-	C	-	T	G	C
Ya’an	SC-2012-L1	T	T	C	A	A	-	C	C	C	C	-	T	-	T	G	C	-	T	C	-	A	C	T	T	G	C
	SC-2012-L2	T	T	C	A	A	C	C	C	C	C	-	T	-	T	G	C	-	T	C	-	A	C	T	T	G	C
Leshan	SC-2013-I1	T	T	C	A	A	C	-	C	T	C	T	-	-	T	G	C	-	-	C	T	-	C	-	T	G	C
	SC-2013-I2	T	T	C	A	A	C	-	C	T	C	T	-	-	T	G	C	-	-	C	T	-	C	-	T	G	C
Korea	GU181244	A	C	T	T	G	T	T	T	G	T	C	T	T	C	A	T	T	A	T	G	G	T	C	C	A	T
**District**	**Name**	**Nucleotide substitutions**																									
		**5**	**5**	**5**	**5**	**5**	**5**	**5**	**5**	**5**	**5**	**6**	**6**	**6**	**6**	**6**	**6**	**6**	**6**	**6**	**6**	**6**	**6**	**6**	**6**	**6**	
		**2**	**3**	**3**	**5**	**6**	**6**	**7**	**8**	**8**	**9**	**0**	**0**	**1**	**1**	**1**	**3**	**3**	**3**	**5**	**5**	**5**	**6**	**6**	**7**	**9**	
		**8**	**1**	**7**	**2**	**0**	**1**	**9**	**3**	**5**	**4**	**3**	**6**	**2**	**5**	**8**	**3**	**6**	**9**	**2**	**4**	**7**	**3**	**6**	**8**	**0**	
Meishan	SC-2011-A1	-	-	C	T	G	T	T	-	-	-	C	-	G	-	T	T	A	T	T	G	G	C	T	G	T	
	SC-2011-A2	-	-	C	T	G	T	T	-	-	-	C	-	G	-	T	T	A	T	T	G	G	C	T	G	T	
Mianyang	SC-2011-B1	-	-	C	T	G	T	T	C	G	-	C	C	G	-	T	T	A	T	T	G	G	C	T	G	T	
	SC-2011-B2	-	-	C	T	G	T	T	C	G	-	-	C	G	-	T	T	A	T	T	G	G	C	T	G	T	
Dazhou	SC-2011-E	T	C	C	T	G	T	T	-	-	-	C	-	G	-	T	T	A	T	T	G	G	C	T	G	T	
Ziyang	SC-2011-F	-	-	C	T	G	T	T	C	G	-	C	-	A	T	T	T	A	T	T	G	G	C	T	G	T	
Chengdu	SC-2012-C1	-	-	C	T	G	T	T	C	G	-	C	-	A	T	T	T	A	T	T	G	G	C	T	G	T	
	SC-2012-C2	-	-	C	T	G	T	T	C	G	-	C	-	A	T	T	T	A	T	T	G	G	C	T	G	T	
Deyang	SC-2012-D1	-	-	C	T	G	T	T	-	-	-	C	-	A	G	T	T	A	T	T	G	G	C	T	G	T	
	SC-2012-D2	-	-	C	T	G	T	T	-	-	-	C	-	G	-	T	T	A	T	T	G	G	C	T	G	T	
Suining	SC-2012-G	-	-	C	T	G	T	T	C	G	C	C	-	A	G	T	T	A	T	T	G	G	C	T	G	T	
Zigong	SC-2012-J	T	C	C	T	-	T	T	-	G	-	A	T	G	-	T	T	A	-	T	G	G	C	T	G	T	
Luzhou	SC-2012-K	T	C	C	T	-	T	T	-	G	-	C	T	G	-	T	T	A	T	T	G	G	C	T	G	T	
Yibin	SC-2012-H1	-	-	C	T	G	T	T	-	-	C	A	T	A	T	T	T	A	-	T	G	G	C	T	G	T	
	SC-2012-H2	-	-	C	T	G	T	T	C	G	C	A	T	A	T	T	T	A	T	T	G	G	C	T	G	T	
Ya’an	SC-2012-L1	T	C	C	T	G	T	T	-	-	C	A	T	G	-	T	T	A	T	T	G	G	C	T	G	T	
	SC-2012-L2	T	C	C	T	G	T	T	-	-	C	A	T	G	G	T	T	A	T	T	G	G	C	T	G	T	
Leshan	SC-2013-I1	-	-	C	T	G	T	T	C	G	C	A	T	A	T	T	T	A	-	T	G	G	C	T	G	T	
	SC-2013-I2	-	-	C	T	G	T	T	-	G	C	A	T	A	T	T	T	A	-	T	G	G	C	T	G	T	
Korea	GU181244	A	T	T	A	A	C	C	A	A	T	T	A	T	A	C	C	G	C	C	T	A	T	A	A	A	

## Discussion

Based on the availability of an effective and feasible RT-PCR diagnostic tool for PToV, a survey of PToV was performed across 12 districts in Sichuan Province Southwestern China. This RT-PCR methodology had been previously proved to be sensitive and specific for PToV [25]. RT-PCR displayed that PToV infections were widespread in large-scale breeding bases and on rural farms in Sichuan. The overall positive rate was 37.96%, and six districts, Suining, Deyang, Yibin, Chengdu, Mianyang and Meishan, had higher infection rates than this. These results may be due to the increased urban agglomeration and greater levels of prosperity within these districts leading to greater movement of, and poorer storage and feeding of livestock with in these areas. According to the PToV infection rates at different growth stages, piglets of 1 to 3 weeks of age were more vulnerable to PToV than the others. Nonetheless, the result is likely to be an incomplete reflection the total number of infections in Sichuan province. The reason for this is that the highest prevalence and greatest anti-PToV Ig G titers were observed in adults [17]. In contrast, the lowest prevalence and lowest anti-PToV Ig G levels were found among 3-week-old piglets [17]. Both ELISA reactivity values and rates of seroprevalence rose markedly in piglets from 3 to 11 weeks [17]. These results suggested that RT-PCR cannot reflect the infections completely due to swine developing their own immune response to the virus resulting in an effective cure in some infected adults. Consequently, further immunological methods should be established to develop a more robust serological surveillance of PToV.

According to the co-infection rates, we can deduce PToV-infected piglets were probably always co-infected with PEDV, RVA and TGEV. Meanwhile, its interesting to note that diarrhea samples from Luzhou tested positive for PToV alone, agreeing with a previous study showing the same results when a survey for enteric pathogens in diarrheic pigs was carried out [26]. However, we cannot make any conclusions about the association between PToV and other enteric pathogens, because of the difficulty of growing the virus in cultured cells.

The comparison of the nucleotide and deduced amino acid sequences of the full-length PToV M nucleotide sequence confirmed that the Sichuan PToV strains were highly conserved for the M gene and most mutations were silent. The phylogenetic tree showed that the 19 Sichuan strains and 2 Korean strains belonged to genotype I, while all other strains resided in genotype II. One special case was that 4 PToV strains within genotype I from the study were classified with 2 strains from Korea in genotype B. Therefore, we speculated that the 4 Chinese PToV strains in subroup B may have originated in Korea, but further study on the origin of circulating PToV in China should be assessed in the future.

Torovirus is widely depicted as a pathogen which is responsible for diarrheal illnesses in animals and human beings [27-29]. In recent years, studies conducted on pigs farmed in Spain, reported that the seroprevalence against PToV in piglets over 11 weeks of age was > 99% indicating that PToV was endemic in piglets in Spain [17]. Epidemiological data on PToV has also been reported from in other countries [3,16], suggesting that it may be circulating in swine populations globally, and with high prevalence. Most PToV infections appear to be subclinical [5], however, there is a potential threat that a much more serious outcome will appear in concurrent infections with other enteric pathogens.

## Conclusion

This is a molecular epidemiological investigation of PToV in Sichuan Province, China. A PToV infection rate of 37.96% (331/872) supported the infectious situation of PToV in southwest China. In addition, nucleotide and amino acid sequence analyses confirmed that the PToV M protein was highly conserved. Since the relationship between PToV infection and diarrhea remains unclear, the virus is still a potential threat for us. Further studies to reveal the epidemiological status of PToV infection in China are essential, and the research should focus on the epidemiology and pathogenic potential of PoTV. Finally, the rapid and continual changes within the torovirus genome highlight the need for persistent surveillance and regular reviews of phylogeny so as to better understand the evolution of this pathogen.

## Consent

Written informed consent was obtained from patients for publication of the data in this manuscript and any accompanying images. A copy of the written consent is available for review by the Editor-in-Chief of this journal.

## Competing interests

The authors declare that they have no competing interests.

## Authors’ contributions

Conceived and designed the experiments: LZ, ZX. Performed the experiments: LZ, YZ. Analyzed the data: LZ, YZ, HW. Contributed reagents/ materials/analysis tools: LZ, HW, YZ, LZ. Wrote the paper: LZ. Checked the grammar: JH. All authors read and approved the final manuscript.
